# Ketosis prone diabetes presenting as fulminant type 1 diabetes

**DOI:** 10.11604/pamj.2018.31.38.16162

**Published:** 2018-09-20

**Authors:** Katerina krompa, Ines Barka, Stéphanie Malard, Sopio Tatulashvili, Camille Baudry, Hélène Bihan, Marinos Fysekidis

**Affiliations:** 1 Service d’Endocrinologie Diabétologie et Maladies Métaboliques, Hôpital Avicenne, 125 rue de Stalingrad, 93009 Bobigny, France; 2 Laboratoire Régional d’Histocompatibilité, Hôpital Saint Louis, 1 avenue Claude Vellefaux, 75010 Paris, France

**Keywords:** Ketosis prone diabetes, fulminant diabetes, diabetes classification

## Abstract

Patients with ketosis prone diabetes have been reported primarily in Africans and African Americans. At presentation, both insulin secretion and insulin action are impaired in ketosis prone diabetes patients. Fulminant diabetes is a subtype of type 1 diabetes reported mainly in the Asian populations characterized by diabetic ketosis or ketoacidosis occurring soon after the onset of hyperglycemic symptoms with inappropriately low HbA1c (< 8.5%). We report here the first case of a ketosis prone diabetes presenting as fulminant diabetes.

## Introduction

Patients with ketosis prone diabetes (KPD) have been reported primarily in Africans and African Americans. At presentation, both insulin secretion and insulin action are impaired in KPD patients. Aggressive diabetes treatment can allow a rapid discontinuation of insulin therapy obtaining a near-normoglycemic remission that may last for a few months to several years [1]. Type 1 diabetes (T1D) is rare in Sub-Saharan populations (4-12/100.00) [2] although epidemiological data is of poor quality. Fulminant diabetes (FT1D) is a subtype of type 1 diabetes reported mainly in the Asian populations characterized by diabetic ketosis or ketoacidosis occurring soon after the onset of hyperglycemic symptoms, high plasma glucose levels (> 2.88 g/l), inappropriately low HbA1c (< 8.5%), low fasting serum C-peptide levels (< 0.3 ng/ml) at onset and no diabetes related autoimmunity markers (absence of anti-GAD and IA2 antibodies) [3].

## Patient and observation

A 62-year-old man of Sub-Saharan African origin was hospitalized with dyspnea, diffuse pain and fatigue. Upon admission, blood pressure was 143/78 mmHg, heart rate 92 beats per minute and physical examination did not show any signs of pulmonary congestion. The patient had been operated on for a gastric adenocarcinoma, had his last chemotherapy thirteen months before the episode of ketosis and was considered in remission. There were no pancreatic or liver abnormalities on the computed tomography (CT) performed during his usual check-up one month before the ketosis incident. Plasma glucose levels were elevated at 3.04 g/l associated with ketoacidosis, arterial blood pH was 7.35 (reference range, 7.36-7.42), ketonemia 7.6 mmol/l, (reference range < 0.5), PCO2 (40 mmHg, reference range, 38-42) and PO2 were normal (99 mmHg, reference range, 70-95). Serum creatinine was at 61 µmol/l (reference range, 59-104, creatinine clearance CKD-EPI formula was at 149 ml/min/1.73m^2^) and liver enzymes were normal. Nutritional status was good, BMI was 20.4 kg/m² and albuminemia 41 g/L (reference range, 36-46). HbA1c was 6.3% (45.4 mmol/mmol, HbA1c measurement is standardized according to the Diabetes Control and Complications Trial). Fructosamine was slightly elevated at 303 mmol/L (reference range, 205-285) and fasting serum C-peptide levels were in the low but normal range at 0.134 ng/ml (reference range, 0.111-1.468).

Adrenal (plasma cortisol was at baseline at 461.5 nmol/l and 60 minutes after synacthen test 620 nmol/l, reference range > 500) and thyroid function (TSH 2.97 mU/L, reference range, 0.27- 4.20) were also normal. Prothrombine time was normal at 96% (reference range: 70-120) and partial thromboplastin time slightly elevated at 1.28 (< 1.20), d dimers were increased and the patient had a CT pulmonary angiography that eliminated pulmonary embolism. CPK were slightly elevated at 397 U/L (reference range, < 190). There was no inflammatory syndrome CRP: 3 mg/L (reference range, < 5), procalcitonin: 0.06 µg/L, (reference range <0.50). Serology for Human Herpes Virus 8, Human Immunodeficiency Virus type 1 and type 2, hepatitis B and C was negative. The patient had normocytic anemia with hemoglobin at 9.9 g/dl (reference range, 12-15), with normal ferritin (140 µg/L, reference range, 30-400), normal B12 levels (399 ng/L, reference range,197-771) and folate deficiency (2.4 µg/L, reference range, 3.9- 26.8). The patient received no transfusion and hemoglobin remained unchanged during his hospitalization. Hemoglobin electrophoresis presented no hemoglobinopathy and glucose 6-phosphate dehydrogenase (12.0 UI/g Hb, reference range, 11-17), enzymatic activity was normal. There was no retinopathy in retinal eye examination and microalbuminuria was negative. Anti-GAD, anti-IA2 and anti-ZnT8 antibodies were negative. The patient was heterozygous for HLA class II alleles DQA1*03:03/DQA1*05:01, DRB1*03/DRB1*13 and DQB1*02/DQB1*03 and the calculated relative risk for a type 1 diabetes was 3.64 [4]. The patient was initially treated with intravenous insulin and later switched to subcutaneous insulin; ketosis resolved 24 hours after admission. Finally insulin was stopped and the patient was discharged on metformin. His diabetes was well controlled 6 months after diagnosis with HbA1c at 6% (42.1 mmol/mmol) on metformin 1000 mg twice a day and fasting serum C-peptide at 0.967 ng/ml.

## Discussion

The fulminant onset of KPD was confirmed from the dissociation of increased fructosamine and the near normal levels of Hb1Ac. HB1Ac levels could have been slightly affected by anemia but there was no hemoglobinopathy in hemoglobin electrophoresis. Oxidative stress has been suggested as a major mechanism in KPD pathophysiology and the presence of G6PD seems to have a protective role since its deficit impacts in the severity of β cell failure [5] and can induce severe acute hemolytic events. Viral involvement with HHV8 infection has been proposed a as the initiating cause of KPD [6] but HHV8 serology was negative in this patient who presented no inflammatory syndrome. This case highlights the fact that ethnicity affects HLA allele prevalence and relative risk calculation for type 1 diabetes may not be appropriate in Sub-Saharan populations since the subjects included in the dataset comprised Caucasian and East Asian populations [4]. This also could explain why the patient presented no T1D related antibodies as markers of autoimmunity. The initial low levels of C-peptide were consistent with low insulin secretion that markedly increased in the six months that followed initial ketosis.

## Conclusion

The near-normoglycemic remission suggested the absence of an ongoing β cell destructive process. To our knowledge this is the first case of a KPD presenting as FT1D and illustrates the role of ethnicity in diabetes classification, prognosis and clinical picture.

## Competing interests

The authors declare no competing interest.

## Authors’ contributions

Katerina Krompa wrote the draft and performed research; Ines Barka performed research participated in the draft and reviewed the article; Stéphanie Malard performed the HLA typing and reviewed the article; Sopio Tatulashvili participated in the draft and reviewed the article; Camille Baudry participated in the draft and reviewed the article Hélène Bihan reviewed the article; Marinos Fysekidis wrote the article and approved the final version of the article. All the authors have read and agreed to the final manuscript.
